# Early-onset neutropenia and mixed phenotype in ADA2 deficiency: diagnostic and therapeutic challenges

**DOI:** 10.3389/fimmu.2025.1639318

**Published:** 2025-10-02

**Authors:** Sibel Kaplan Sarıkavak, Özge Türkyılmaz Uçar, Burcu Cil, Michael S. Hershfield, Teresa K. Tarrant, Pinar Gökmirza, Çiğdem Aydoğmus

**Affiliations:** ^1^ Department of Pediatric Allergy and Clinical Immunology, University of Health Sciences, Istanbul Haseki Training and Research Hospital, Istanbul, Türkiye; ^2^ Department of Pediatric Allergy and Clinical Immunology, Gaziosmanpaşa Training and Research Hospital, Istanbul, Türkiye; ^3^ Department of Pediatric Allergy and Clinical Immunology, University of Health Sciences, Basaksehir Cam and Sakura City Hospital, Istanbul, Türkiye; ^4^ Department of Medicine, Division of Rheumatology and Immunology, Duke University School of Medicine, Durham NC, United States; ^5^ Duke University School of Medicine, Durham, NC, United States; ^6^ Department of Medicine, Division of Rheumatology and Immunology, Duke University School of Medicine, Durham, NC, United States; ^7^ Department of Medicine, Division of Rheumatology, Durham Veterans Administration Health System, Durham, NC, United States

**Keywords:** adenosine deaminase 2 deficiency, hematologic manifestations, neutropenia, hemophagocytic lymphohistiocytosis, hematopoietic stem cell transplantation

## Abstract

**Background:**

Adenosine deaminase 2 deficiency (DADA-2) is an autoinflammatory syndrome caused by mutations in the *ADA2* gene. ADA-2 functions as an enzyme in purine metabolism and is presumed to play roles in immune regulation. The clinical spectrum of DADA2varies widely, from vascular inflammation and immune dysregulation to hematological abnormalities, including pure red cell aplasia and hemophagocytic lymphohistiocytosis (HLH). This study aimed to describe the clinical, demographic, and immunological profiles of seven DADA-2 patients to broaden the understanding of its hematological and immunological manifestations and provide insight for early diagnosis and treatment strategies.

**Methods:**

Data were collected from patient medical records at the Department of Pediatric Allergy and Clinical Immunology, Basaksehir Cam and Sakura City Hospital. The study included genetic analysis, flow cytometry for lymphocyte subpopulations, and ADA-2 enzyme activity measurement.

**Results:**

Seven patients from five families were included, predominantly male, with an average symptom onset at 15 months. Hematological findings were present in all patients, with neutropenia observed at the initial presentation (100%). HLH developed in two patients, contributing to a higher mortality rate of 42.8%. Bone marrow analysis in affected patients revealed hypocellularity and marked T-cell infiltration, with fibrosis detected in one. Despite no evidence of viral triggers (EBV, CMV, VZV, Parvovirus B19), HLH occurred in two patients, suggesting a primary immune dysregulation. Inflammatory and immunodeficiency-related findings were also observed, suggesting a mixed phenotype as the most common presentation. Genotype-phenotype analysis showed that patients with undetectable ADA2 enzyme activity or loss-of-function mutations had more severe hematological involvement. In contrast, a patient with residual enzyme activity exhibited a mixed phenotype. Three patients underwent successful hematopoietic stem cell transplantation (HSCT), reversing disease manifestations.

**Conclusion:**

Our findings reinforce that DADA2 can initially present as isolated neutropenia, and frequently exhibits a mixed phenotype encompassing hematologic, immunologic, and inflammatory features. HLH is a severe complication that may arise without infectious triggers. Genetic testing for *ADA2* should be incorporated into diagnostic panels for congenital neutropenia to avoid delays in diagnosis. Genotype–phenotype correlations offer some prognostic insights, but residual enzyme activity may not fully predict disease severity, underscoring the need for individualized management.

## Highlights

- Early-onset neutropenia may be the first and only presenting sign of ADA2 deficiency, emphasizing the importance of including ADA2 in targeted gene panels for congenital neutropenia.
- ADA2 deficiency can present with overlapping hematologic, inflammatory, immunologic, and even presymptomatic phenotypes within the same family, highlighting significant intrafamilial variability.
- Although genotype–phenotype correlations exist, residual ADA2 enzyme activity by HPLC does not reliably predict disease severity, underscoring the complexity of disease pathogenesis.
- Hemophagocytic lymphohistiocytosis (HLH) is a potentially life-threatening manifestation of DADA2 and may occur without viral triggers, indicating a primary immune dysregulation.

## Introduction

Adenosine deaminase 2 deficiency (DADA-2) is an autoinflammatory syndrome caused by homozygous or compound heterozygous mutations in the *ADA2* gene, first identified in 2014 (1). ADA-2 primarily functions as a catalytic enzyme involved in purine metabolism but also appears to have roles in the immune system (2). ADA-2 is an extracellular enzyme primarily secreted by monocytes and macrophages, with potential contributions from neutrophils, NK cells, and regulatory T cells (3–5). DADA2 causes skewed M1 macrophage development, leading to disrupted endothelial integrity (5, 6). Some DADA2 patients show increased TNF-α, with type I and II IFNs possibly involved (6). Clinical manifestations range from autoinflammation with fever, early-onset strokes, and vasculopathy to immune dysregulation, characterized by hypogammaglobulinemia, lymphoproliferation, and increased susceptibility to infections. Hematological abnormalities, such as pure red cell aplasia(PRCA) and cytopenias, may arise from autoimmune mechanisms or bone marrow failure with marrow aplasia (7). Hemophagocytic lymphohistiocytosis (HLH) has also been reported and can be life-threatening (8, 9). Recently, an International Consensus Statement classified DADA2 into four disease phenotypes for phenotype-specific discussions: inflammatory, hematologic, immunodeficient, and presymptomatic (10). Apart from the presymptomatic group, these phenotypes are not mutually exclusive. Multiple reports also highlight overlapping features among these conditions (11–14). Lee et al. also reported a genotype-phenotype correlation, with absent ADA2 activity linked to red cell aplasia and bone marrow failure, and residual activity associated with vascular phenotypes (15).

Here, we describe the demographic, clinical, and immunological features of seven DADA2 patients, aiming to broaden the hematological and immunological spectrum of the disease and offer insights for early diagnosis and treatment.

## Materials and methods

We collected demographic, clinical, data from patient medical records, which were reviewed in detail. A questionnaire covering demographics, and clinical data (age of symptom onset, age at the diagnosis, follow-up, family history, infections, immunologic tests, vasculitis, inflammation, hematological, and immunologic features, diagnostic tests, treatments, and clinical course) was completed for each patient. Serum immunoglobulin levels were measured via nephelometry, peripheral lymphocyte subgroups were analyzed by flow cytometry, with results compared to reference values for Turkish children and adults (16, 17). Genetic diagnoses were confirmed by Sanger validation. The diagnosis of ADA-2 deficiency was supported by measuring ADA-2 activity, in accordance with the consensus report (10). Plasma spot ADA-2 activity levels (mU/g protein) were measured using dried plasma spots by Michael S. Hershfield, MD, and his team at Duke University. Sağlık Bilimleri University Faculty of Medicine Ethics Committee approved our study (KAEK/14.02.2024.113). Families of each patient provided written informed consent, and all studies were conducted in accordance with the principles of the Declaration of Helsinki. The data analyzed in the study were processed using SPSS 23.

## Results

### Demographic and clinical findings

Seven patients from five families were included in the study, comprising 1 female and 6 males. The average age of symptom onset was 15 months (range: 1–84 months), the average age of diagnosis was 42 months (range: 3–108 months), and the average current age is 4.5 years (range: 1–8 years). Three of the patients deceased at the ages of 2, 3, and 11 years. Consanguineous marriage was present in three families. All patients were of Turkish origin, except for one family in which all three siblings with DADA2 were of Syrian origin. Except for the siblings P5.1, P5.2, P5.3 the families had no similar disease history. The P3 had a history of two siblings who died in the neonatal period due to unknown causes. The remaining patients had no siblings. The main findings observed at the first presentation and the onset of the disease were neutropenia (100%), recurrent infections (71,5%), lymphopenia (71,5%).

At the end of the study, P1, P2, P5.2, P5.3 were still alive, and the mean follow-up duration for all patients was 3.75 years (min-max: 1-7). P1, P2, P5.2 are being followed up after undergoing bone marrow transplantation. P3 passed away at the age of three due to HLH and sepsis while awaiting screening for bone marrow transplantation. P4 received only two doses of etanercept; but a hemorrhagic necrosis appeared on her extremities and due to uncontrolled inflammation or infection, the patient deceased at the age of 11. P5.1 passed away at the age of 2 due to sepsis following ecthyma gangrenosum. P5.3 is currently under follow-up and remains asymptomatic, with no history of recurrent infections.

#### Hematologic manifestations

Hematologic involvement was observed in all patients. Upon admission, all patients presented with neutropenia (100.0%), five patients (71.5%) had lymphopenia, and four patients (57.0%) had anemia (**Tables 1**, **2**). Four patients (P1, P2, P4, P5) received G-CSF treatment at a dose of 10 mcg/kg/dose during episodes of febrile neutropenia; however, no increase in neutrophil count was observed. While pure red cell anemia was the initial clinical presentation in P1 and P5.2, P2 and P3 developed anemia during follow-up. P5.3’s anemia improved with iron therapy. P1, P2, and P3 all exhibited lymphadenopathy and hepatosplenomegaly during follow-up. Bone marrow analysis in P1, P4, P5.1, and P5.2 revealed no malignancy but showed hypocellularity in the myeloid lineage. In P2, the bone marrow was hypocellular with sparse, scattered myeloid elements, erythroid hyperplasia with left shift, and increased megakaryocytes, consistent with congenital neutropenia. A marked increase in T lymphocytes (25–27%) within the intertrabecular spaces was noted, raising suspicion for a lymphoproliferative disorder. Similarly, in P3, erythroid hyperplasia, reduced myeloid cells, and fibrozis were observed, along with elevated interstitial T lymphocytes (18–20%). These cells showed balanced CD4/CD8 expression, and no evidence of lymphoma infiltration was detected. The T-cell lymphocytosis in both cases warrants further evaluation for underlying lymphoproliferative disease. P2 and P3 later developed persistent and prolonged fever, alongside anemia and thrombocytopenia. Given the elevated ferritin levels, hypertriglyceridemia, elevated liver enzymes, pancytopenia, and hepatosplenomegaly, bone marrow aspiration was conducted again, revealing hemophagocytosis. Consequently, the HLH treatment protocol was initiated for P2, and P3. Viral screening conducted during febrile episodes, cytopenias, and HLH showed no evidence of EBV, parvovirus B19, CMV, or VZV infection.

**Table 1:** Demographic features of ADA-2 deficieny patients.

	P1	P2	P3	P4	P5.1	P5.2	P5.3
Gender	Male	Male	Male	Female	Male	Male	Male
Current age (yrs)	8	6	Deceased at age of 3	Deceased at age of 11	Deceased at age of 2	3	1
Age at onset of symptoms (mo)	1	1	5	84	11	1	1
Age at diagnosis (mo)	40	66	30	108	–	5	3
Consanguinity	–	–	+	+	+	+	+
Dead sibling	–	–	+	–	–	+	+
Initial symptoms and laboratory analysis	PRCA, Recurrent infection and fever, neutropenia	Recurrent infection and fever, Neutropenia	Recurrent oral aphthous lesions, Recurrent infection and fever, recurrent gastroenteritis, Anemia,neutropenia,	Recurrent oral aphthous lesions, skin abscesses, recurrent gastroenteritis,	Recurrent oral aphthous lesions, recurrent pneumonia,skin abscesses	PRCA, ADA-2 deficent brother, PRCA,	ADA-2 deficent brother,
Hematological	neutropenia, PRCA, lymphopenia	neutropenia, lymphopenia, thrombocytopenia	anemia, neutropenia, thrombocytopenia	neutropenia, lymphopenia	neutropenia, lymphopenia,	neutropenia, lymphopenia, anemia	neutropenia, anemia
Immunological	Low IgA, IgG, IgM	Low B cells	Low IgA, IgG, IgM Low B cells	Normal Ig levels, candida esophagitis, fungal pneumonia.	Normal	Low IgA, IgG, IgM	Low IgA, IgG, IgM
Vasculitis/inflammation				Recurrent oral aphthous lesions, skin abscesses	Recurrent oral aphthous lesions, skin abscesses,		
Neurological	–	–	–	–	–	–	–
HSM/Lympadenopathy/HLH	+/+/-	+/+/+	+/+/+	-/-/-	-/-/-	-/-/-	-/-/-
Treatments	IVIG, TMP-SMX, G-CSF, HSCT	IVIG, TMP-SMX, G-CSF, HSCT HLH protocol	IVIG, TMP-SMX HLH protocol	G-CSF, anti-tumornecrosis factor (TNF)-α (etanercept)	G-CSF	HSCT	–
Prognosis	Alive	Alive	Exitus	Exitus	Exitus	Alive	Alive
Genetic mutation	ADA2(Exon 2, c.144_145insG (p.Arg49Alafs*13), Exon 4, c.629delT (p.lle210Thrfs*57)), compound heterozgyte	ADA2, Exon 9, c.1271A>G (p.His424Arg), homozygous,	ADA2, Exon 2, c.140G>T (p.Gly47Val), homozygous	ADA2 (c.G962A; p.G321E), homozygous	ADA2, Exon 3, c.506G>A (p.Arg169Gln),homozygous (diagnosis was retrospectively assumed based on the patient’s clinical features and family history.	ADA2, Exon 3, c.506G>A (p.Arg169Gln),homozygous	ADA2, Exon 3, c.506G>A (p.Arg169Gln), homozygous
Plasma spot ADA-2 activity, mU/g protein (130 ± 53)	–	0.0 (130 ± 53)	4.4 (130 ± 53)	–	–	0.0 (130 ± 53)	–
Consequence	Del/ins_frameshift and non-sense	Missense	missense	missense	–	missense	missense
ACMG classsification	Pathogenic	VUS	Pathogenic		–	Pathogenic	Pathogenic

+: present, -: not present, Ig, immunoglobulin, PRCA, pure red cell aplasia, HSM, hepatosplenomegaly; HSCT, hematopoietic stem cell transplantation; ADA2, adenosine deaminase 2; G-CSF, granulocyte colony-stimulating factor

**Table 2:** Laboratory analysis of adenosine deaminase-2 deficiency patients.

	P1 (40months old)	P2 (66 moths old)	P3 (30 months old)	P4 (108 months old)	P5.1 (12 months old)	P5.2 (5 months old)	P5.3 (3 months old)
Leukocyte (/mm³)	**3500**	**1900**	**2600**	**1800**	**1350**	**3600**	6400
Hemoglobin (g/dL)	**5.9**	10.7	10.8	10.5	9.8	**6.8**	**7.8**
Thrombocyte (x10^9^/L)	231	275	214	225	233	**181**	407
Neutrophil(/mm³)	**1200**	**10**	**330**	**500**	**10**	**570**	**1300**
Lymphocyte(/mm³)	**970**	**1200**	2080	**800**	**1300**	**1700**	4000
IgG(mg/dl)	**363**	1247	**322**	1210	751	**306**	**286**
IgA(mg/dl)	**14**	159	**22**	122	90	**16**	**6**
IgM(mg/dl)	**27**	112	**11**	234	46	**25**	**12**
IgE(IU/mL)	13.4	148	2	12	27	26,7	3.87
CD3	**649.9**	1140.0	1747.2	**584.0**	1001.0	1377.0	2400.0
CD4	**397.7**	**468.0**	1144.0	**384.0**	728.0	986.0	2960.0
CD8	**261.9**	**192.0**	811.2	**192.0**	351.0	629.0	880.0
CD19	**242.5**	**31.2**	187.2	**144.0**	221.0	**255.0**	1320.0
CD16-56	**48.5**	120.0	603.2	**112.0**	195.0	**251.6**	**232.8**
CD19+CD27+ıgD-		81.6	59.9			16.8	15.6
CD4+CD31+CD45RA (%)	–	**39**	**37**			60	70

Ig, Immunoglobulin; CD, Cluster of Differentiation. Low counts are shown in bold.

### Immunodeficiency manifestations

All patient had recurrent neutropenic infection except P5.2, and P5.3. Among the three patients who died, P4, P5.1 had skin abscesses and recurrent gastroenteritis. P4’s clinical history also included candida esophagitis, gastritis, and fungal pneumonia. P1, P3, P5.2, and P5.3 had hypogammaglobulinemia. P1, P2, P4, and P5.2 had low B cell counts. CD3 T cell counts were reduced in P1 and P4. Both CD4 and CD8 T cell counts were below the age-appropriate reference ranges in P1, P2, and P4. Reduced recent thymic emigrant (RTE; CD4+CD31+CD45RA+) percentages were observed in P2 and P3. Lastly, low NK cell (CD16-56) counts were noted in P1, P4, P5.2, and P5.3. Three patients (P1, P2, and P3) received intravenous immunoglobulin (IVIG) and trimethoprim-sulfamethoxazole prophylaxis due to both frequent infections and their immunological findings. Detailed immunological evaluations of the patients are presented in **Table 2**.

### Inflammatory and/or vasculitic manifestations

All patients had recurrent fever and elevated sedimentation rate and CRP, except P5.2 and P5.3. P3, P4, and P5.1 experienced recurrent oral aphthous lesions. P4’s clinical history included recurrent skin abscesses and hemorrhagic necrosis on the leg and foot. Etanercept was initiated for P4, but the patient died due to uncontrolled inflammation. Patient P5.1, who had a history of skin abscesses, later developed an indurated lesion extending from the right inguinal area to the perineum with central ulceration, suspected to be ecthyma gangrenosum. Congenital neutropenia was initially suspected, but a congenital neutropenia panel revealed no mutations. Whole exome sequencing (WES) was planned; however, the patient unfortunately passed away before it could be conducted. It was later discovered that two of the patient’s siblings had DADA2, confirming that P5.1 also had DADA2. None of our patients had a history of stroke, neuropathy, or encephalitis (**Table 1**).

Comprehensive tests for ANA, Anti-dsDNA (IFA), Nucleosome, Anti-histone antibody, Anti-Scl 70, Anti-DFS70, Anti-SSA, Anti-SSB, Anti-Ro-52, Anti-Sm, Anti-Sm/RNP, Anti-Ku, Anti-Mi-2, Anti-CENP B, Anti-PM/Scl, Anti-PCNA, Anti-mitochondrial antibody (AMA), Anti-Jo1, anti-ribosomal P protein, ANCA, C3, and C4 were conducted as part of a general screening for autoimmunity. Follow-up results for P2 showed Anti-Ro-52 (+++), Anti-SSA (+), Anti-DFS (+), and Anti-mitochondrial antibody (++). In P3, ANA with a titer of 1/320 in a speckled pattern was detected. All tests were negative in the other patients (not shown in table).

#### Presymptomatic manifestations

Although anemia and neutropenia were detected in P5.3 at the time of admission, the patient’s anemia responded to iron therapy and has been monitored without frequent infections.

#### Genetic analysis and plasma spot ADA-2 activity

Among the six patients with identified genetic mutations, five had missense mutations, whereas P1 had a del/ins frameshift and nonsense mutation. Five of these mutations were classified as pathogenic, while P2 had a variant of uncertain significance (VUS) mutation; however, measurement of ADA-2 enzyme levels supported the diagnosis. Plasma spot ADA-2 activity was measured at 0.0 mU/g protein in P2 and P5.2, and 4.4 mU/g protein in P3. In P5.1, ADA-2 mutations or activity levels had not been studied or identified. However, when ADA-2 mutations were later identified in two of the patient’s siblings, combined with clinical findings, it was concluded that the diagnosis was DADA2 (**Table 1**).

## Discussion

In this study, we present clinical, genetic, and immunologic data from seven patients with ADA2 deficiency, with a notable predominance of hematologic findings—particularly neutropenia—as the initial presentation. Consistent with previous literature, the majority of our patients presented in early childhood, with a median age of 4.5 years (9, 18). Although some studies reported a balanced sex ratio, we also observed male predominance, consistent with others (9, 18). The high rate of consanguinity (3 of 5 families) and sibling clustering underscores the autosomal recessive inheritance pattern and supports recommendations for family screening, particularly in populations with high consanguinity rates. Our study also supports phenotypic variability among siblings with identical mutations, including presymptomatic cases, reinforces the need for a high index of suspicion and longitudinal monitoring (9, 18–20).

The distinction between vasculitic and hematologic phenotypes is well recognized in DADA2, but many patients—like ours—present with a mixed clinical picture (9–11, 13, 15, 18). Although certain studies emphasize primarily inflammatory and neurologic manifestations, a recent review highlighted hematologic features as more prevalent—consistent with our findings (11, 13, 15, 18, 21). Patients are generally older in studies with predominant inflammatory features (13). In contrast, the median age for hematologic manifestations is around 0.5 years, possibly explaining the higher rate of hematologic findings in our cohort (13).

Neutropenia was present in all patients at the time of admission in our study. This contrasts with larger cohorts where anemia (25.6%) has been identified as the most prevalent hematologic abnormality, and neutropenia is reported in only 13–20% of cases (9, 21). Similar to previous case reports, our findings suggest that ADA2 deficiency should be strongly considered in pediatric patients with unexplained or treatment-resistant neutropenia (22, 23). Importantly, current neutropenia gene panels do not routinely include ADA2, which may contribute to delayed diagnosis and missed cases. In our cohort, one patient could not be diagnosed through a neutropenia gene panel due to the absence of ADA2 gene in the panel and was only diagnosed following genetic confirmation in a symptomatic sibling. Diagnostic delays in other patients further underscore the need to incorporate ADA2 gene into targeted neutropenia panels.

In our study, bone marrow analysis also revealed fibrosis, along with increased T cells, consistent with previously reported findings of lymphohistiocytic aggregates and T-cell infiltration in DADA2 patients (13, 22, 24). Two patients developed HLH, one of whom died, highlighting its potentially life-threatening course (9). Although HLH is recognized in ADA2 deficiency, its classification as primary or secondary remains debated. While viral triggers such as EBV, parvovirus B19, CMV, and VZV have been implicated, routine viral screening in our cohort during febrile, cytopenic, and HLH episodes showed no evidence of these infections (8). This supports a primary immune dysregulation mechanism in DADA2-associated HLH in some cases (25).

In addition to hematologic findings, inflammatory signs—including oral aphthous ulcers, recurrent fever, skin abscesses, necrosis, elevated ESR/CRP, and ANA positivity—were also noted, in line with findings from other studies (18, 26). In contrast to previous reports, no neurological involvement was observed in our cohort (18). Our study also demonstrated decreases in various immunological parameters, including low B cell counts, as well as decreased CD4, CD8, RTE, and NK cell levels in DADA2 patients (27–29). Three patients with hypogammaglobulinemia, and decreased B cell counts, and recurrent infections were treated with IVIG and prophylactic antibiotics.

The relationship between genotype and phenotype in DADA2 remains complex. In our cohort as well, the identified ADA2 mutations were distributed across various exons, with no apparent clustering within specific genomic regions. Several of the mutations identified by Lee et al. as having genotype–phenotype correlations were also observed in our cohort, with clinical features aligning with those reported in their study (15). Notably, two mutations identified in our P1—R49Afs*13 and I210Tfs*57—have been associated with bone marrow failure and PRCA-only phenotypes, respectively. In P3, the G47V mutation was detected, which has been linked to both vasculitis and bone marrow failure. Additionally, the R169Q variant, identified in patients P5.1, P5.2, and P5.3, has been associated with a broad phenotypic spectrum, including stroke, red cell aplasia, and profound cytopenias. Interestingly, these three siblings exhibited distinct clinical presentations, further highlighting the phenotypic variability even among individuals carrying the same mutation (15).

Another finding supporting the genotype–phenotype correlation is that our patients with severe hematologic involvement exhibited either undetectable ADA2 enzyme activity (0.0 mU/g; P2, P5.2) or harbored frameshift/nonsense mutations (P1). Conversely, P3, who carried a missense mutation and presented with both hematologic and vasculitic features, had a residual enzyme activity of 4.4 mU/g. This aligns with previous reports indicating that missense mutations with partial enzymatic function are often linked to vasculitis-predominant phenotypes (15). However, the presence of hematologic findings in this patient—representative of a mixed phenotype—suggests that enzyme activity alone may not fully account for the clinical heterogeneity observed in DADA2. Notably, in our study, ADA2 activity was measured using high-performance liquid chromatography (HPLC), rather than via *in vitro* functional assays using recombinant protein (15). Therefore, results should be interpreted with caution, particularly when attempting to correlate enzyme activity levels with clinical phenotype.

Mortality was also observed in our cohort of DADA2 patients (9, 18). While TNF inhibitors are the mainstay treatment for vasculitic and inflammatory forms, they are less effective in hematologic or immunodeficient cases, where hematopoietic stem cell transplantation (HSCT) remains the only curative option (15, 18, 30). Three of our patients successfully underwent HSCT with favorable outcomes, in line with previous reports showing reversal of disease manifestations post-transplant. Nonetheless, HSCT carries risks, optimal timing remains a key clinical challenge (12, 30).

In conclusion, although based on a limited number of patients, our study provides valuable insights into the diverse clinical spectrum of DADA2—including hematologic, immunologic, inflammatory, and presymptomatic phenotypes, most often presenting in mixed form—with early-onset neutropenia observed in all cases. The findings underscore the complexity of genotype–phenotype correlations, as even identical mutations were associated with variable clinical manifestations. Although some DADA2 patients exhibit well-defined clinical phenotypes, the presence of mixed phenotypes in others suggests that enzyme activity alone may not fully account for the clinical heterogeneity observed in the disease. Furthermore, the occurrence and fatal potential of HLH in DADA2 highlight the need for heightened clinical awareness and timely intervention. Finally, our experience supports the efficacy of hematopoietic stem cell transplantation as a curative option in selected patients with hematologic or immunodeficient phenotypes.

## Data Availability

The raw data supporting the conclusions of this article will be made available by the authors, without undue reservation.
